# Supplementary material for: Ethical considerations in the prehospital treatment of out-of-hospital cardiac arrest: A multi-centre, qualitative study
Source: PLoS One. 2023 Jul 26;18(7):e0284826. doi: 10.1371/journal.pone.0284826 (PMC10370897; doi:10.1371/journal.pone.0284826)
Supplement: S2 File — (PDF) [file pone.0284826.s002.pdf]

Appendix 2: Translated interview guide

Categories	Explanation	Questions
**Experience**	The level of experience of the participants	• How long have you worked as a prehospital physician?<br>• What are your sub-speciality in anaesthesiology besides prehospital medicine?
**Decision-making process**	Open reflection on factors playing into decision-making<br><br>**<u>AWARENESS</u>** on areas that needs further probing!	• Try telling me about the cardiac arrest situation we experienced today as you remember it.<br>• What considerations did you make in relation to the cardiac arrest?<br>• What factors played a part in your final decision? *Was it one single factors that made you decide?*<br>• Would you have done anything differently? *If yes, why? If no, why not?*<br>• How did it feel to make the decision?<br>• How do you experience withholding resuscitation compared to terminating already initiated resuscitation?<br>• Who were involved in your final decision?
**Individual factors**	Other factors that may have an influence provided that they haven't been mentioned in the answers to the questions above.	• What part do the patient's age play?<br>• Can you describe an OHCA situation you experienced as difficult or challenging?<br>• Are there any groups of patients who complicate a cardiac arrest situation? *If yes, which?*<br>• What part do relatives play for you?<br>• What are your opinions on advance directives or DNACPR? *Both verbal and in writing.*
**Closing questions**	The experience of ethics and ethical challenges	What are your understanding of ethics in prehospital treatment?<br>How do you use your understanding in your work?<br><br>*Do you have anything to add? Something we haven't touched upon?*
