# Supplementary material for: Ethical considerations in the prehospital treatment of out-of-hospital cardiac arrest: A multi-centre, qualitative study
Source: PLoS One. 2023 Jul 26;18(7):e0284826. doi: 10.1371/journal.pone.0284826 (PMC10370897; doi:10.1371/journal.pone.0284826)
Supplement: S3 File — (PDF) [file pone.0284826.s003.pdf]

Appendix 3: Translated observation and field note guide

Category	Includes	Additional registrations
Appearance	• Clothes<br>• Age<br>• Gender<br>• Physical appearance	Profession, social status, socioeconomic level (estimated), religion, ethnicity
Verbal interac-tions and behav-iour	• Who talks with who<br>• For how long<br>• Who initiates the con-versation<br>• Language or dialect<br>• Tone of voice	- Clothes<br>- Age<br>- Gender<br>- Physical appearance<br>^The one speaking<br>Dynamic of the interaction<br><br>How much of the conversations concern other things than medical/clinical topics?<br>**Attention on ethical challenges**: Are they ad-dressed? Who initiates conversations on ethics (relatives, EMT/paramedic/ physician/others? How is the prompt received?
Physical behav-iour and interac-tion	• What do people do?<br>• Who does what?<br>• Who interacts with who?<br>• Who does not interact with anyone?	How do people use their body language and tone of voice to communicate potential feelings? What actions do people do to indicate potential feelings towards each other, social rank or pro-fession?
Personal space	• How close are people to each other?	What do the individuals' personal space prefer-ences mean for the interaction?
Human traffic	• People who enter, exit or are present (**ONE SCENE**)	Where do people leave and exit from? How long do they stay? Who are they? (ethnicity, age, gen-der)? Are they alone or together with someone? **Number of people**
Noticeable people	• Identification of peo-ple who receive a lot of attention from oth-ers	Characteristics of noticeable individuals: What differentiates them from others? Are people ap-proaching them?<br>**How does the physician appear? Approacha-ble? Open to suggestions?**

REMEMBER: Objective descriptions!
Document: Events and conversations, note body language, ambience, attitudes, the environment in general, and interactions between participants. OTHER RELEVANT INFORMATION **(e.g. my perception of situations, subjective analysis)**

REMEMBER: Drawings of the observation localisation (setting)
